# AI-driven educational reform: enhancing talent cultivation in computer-related majors for the digital era

**DOI:** 10.3389/fpsyg.2026.1790916

**Published:** 2026-09-07

**Authors:** Jun Wang, Pengfei Li

**Affiliations:** College of Computer Science and Technology, Shenyang University of Chemical Technology, Shenyang, China

**Keywords:** AI-driven education, educational reform, intelligent assessment, interdisciplinary integration, talent cultivation

## Abstract

The rapid development of artificial intelligence (AI) has driven the emergence of new quality productive forces, reshaping talent demands in the digital era. In response to this transformation, this study explores a comprehensive reform of the talent cultivation model in computer-related majors, deeply integrating AI technologies into the curriculum, teaching methods, and practical training. Grounded in the “new engineering” educational philosophy, the proposed model emphasizes interdisciplinary integration, personalized learning, and collaborative education, aligning with the education, science, and talent strategy outlined in the 20th National Congress of the Communist Party of China. This strategy highlights the importance of using education to drive technological innovation and cultivate high-quality talent with innovative thinking and practical skills. The reform framework includes course restructuring, smart teaching integration, interdisciplinary collaboration, practice-oriented learning enhancement, and a comprehensive evaluation system. This model uses the comprehensive evaluation system to systematically assess students’ technical proficiency, innovation awareness, and practical problem-solving skills, providing data-driven insights to support educational reform and optimize teaching strategies. The reform framework has been phased in across four undergraduate programs, aiming to establish a replicable and scalable talent cultivation model under the paradigm of AI-driven educational transformation. Preliminary results show significant improvements in students’ technical capabilities, innovation capacity, and readiness to solve real-world challenges.

## 1 Introduction

The rapid development of artificial intelligence (AI) has profoundly transformed industrial production, social structures, and talent cultivation systems. As a key driver of new productive forces, AI is reshaping labor market demands and redefining the core competencies required of future professionals, particularly in computing and related disciplines (Alharbi, 2024). However, traditional teaching models, fragmented resource allocation, and single knowledge transmission methods have been unable to adapt to the rapid advancements of emerging technologies and the changing needs of society, leading to a widening gap between talent supply and societal demands (Qian et al., 2025; Lakshmi et al., 2023). Consequently, higher education institutions must reform traditional teaching paradigms to foster innovative, interdisciplinary, and application-oriented talents capable of thriving in an AI-driven era.

As one of the disciplines most profoundly influenced by artificial intelligence, computing stands at the forefront of educational transformation (Liu, 2024). It faces a dual challenge: on one hand, it must keep pace with the rapid technological advancements to ensure students are equipped to tackle emerging technologies; on the other hand, the traditional educational system has significant structural limitations, particularly in terms of curriculum updates, innovative teaching methods, and interdisciplinary collaboration, which have failed to keep up with industry needs (Reihanian, 2025; Wang, 2023; Hwang et al., 2020). This disconnect hinders students’ innovative thinking and practical abilities, preventing them from fully developing the comprehensive skills necessary to address complex problems (Papagiannis and Pallaris, 2024). To address these challenges, higher education must undergo a profound transformation, promoting the integration of education, technology, and talent cultivation, achieving cross-disciplinary fusion and collaboration, and ensuring that the education system is closely aligned with industry demands (Du, 2025). In response to these challenges, this paper presents a systematic reform initiative that aims to redesign the talent cultivation model for computer science and related majors within the broader context of innovation-driven productivity. Anchored in AI technologies and new engineering education philosophies, the initiative focuses on integrating intelligent tools throughout the teaching process, establishing personalized and adaptive learning paths, enhancing interdisciplinary curriculum design, and building strong university–industry collaboration mechanisms. By developing an intelligent evaluation system and a smart teaching environment, the study seeks to bridge the gap between higher education and industrial innovation, thus preparing students to become future-oriented, high-level talents with strong innovation capability, practical skills, and ethical awareness.

This study introduces the reform framework, implementation strategies, and preliminary outcomes of the initiative, with a focus on the innovative integration of AI technologies into the curriculum, pedagogy, and practical training processes. The initiative is designed to serve as a practical and replicable model for similar reforms in engineering and technology education across institutions. Specifically, it introduces three key innovations:

(1) Conceptual Innovation

This study presents a forward-looking conceptual framework for AI-integrated education, which emphasizes the synergistic interaction between education, technology, and talent cultivation. This framework aligns with the evolving demands of the digital economy, fostering an educational ecosystem that not only adapts to but actively shapes the future workforce’s competencies, ensuring the continuous alignment of educational goals with industry needs.

(2) Methodological Innovation

Methodologically, this study proposes a novel talent cultivation model that integrates interdisciplinary collaboration and personalized learning pathways. This model emphasizes a collaborative approach between academia and industry, aiming to foster innovation-driven, application-oriented talent capable of meeting the complex demands of modern industries. It involves restructuring the curriculum to bridge the gap between theoretical knowledge and real-world problem-solving skills.

(3) Technological Innovation

Technologically, the initiative leverages advanced AI tools—including intelligent assessment systems, adaptive learning platforms, and real-time feedback mechanisms—to enhance the precision, effectiveness, and adaptability of the teaching process. These technologies facilitate continuous, data-driven assessment of student progress, enabling personalized interventions and improving overall teaching efficacy. By integrating AI into the learning environment, the system fosters a dynamic feedback loop that empowers students to develop both technical and innovative skills.

## 2 Literature review

### 2.1 Overview of global trends in engineering education reform

The reform of engineering education has long been a critical focus in global higher education, particularly in light of the rapid technological advances driven by AI, big data, and automation. Numerous studies have emphasized the need to align higher education curricula with emerging industry demands and evolving skillsets required in the digital era (Aljohani et al., 2022; Singun, 2025). Early reform initiatives were primarily concerned with updating engineering curricula to reflect technological advancements such as computerization and automation. However, in the past decade, the rise of advanced technologies has triggered a new phase of transformation characterized by the convergence of technology, pedagogy, and innovation ecosystems (Wang et al., 2024; Tan et al., 2025).

Some internationally recognized frameworks, such as the Conceive-Design-Implement-Operate (CDIO) approach and ABET accreditation standards, have played a pivotal role in redefining engineering education. These frameworks not only focus on the transmission of technical knowledge but also emphasize the development of comprehensive competencies, such as creativity, critical thinking, and ethical awareness (Crawley, 2007; Akera, 2019). The CDIO framework, through its systematic engineering practice process, enables students to develop a comprehensive set of skills, from conception to implementation, emphasizing innovative thinking, teamwork, and interdisciplinary integration (Crawley, 2007; Tanveer and Usman, 2022). ABET accreditation standards, on the other hand, require engineering curricula to include not only fundamental technical and scientific knowledge but also to cultivate communication skills, leadership, and the ability to solve complex engineering problems. This shift represents a significant move from traditional knowledge delivery to an education model focused on competencies (Raj, 2022).

This transformation reflects a profound change in global engineering education philosophies. With societal needs changing, especially in response to rapid developments in digitalization, globalization, and social responsibility, traditional technology-centered engineering education can no longer meet modern societal demands (Lyngdorf et al., 2024). New frameworks advocate for the enhancement of engineers’ comprehensive abilities through interdisciplinary integration, ensuring that they are equipped not only with technical skills but also the flexibility to address diverse and complex global challenges (Beldad and Miedema, 2025).

Building upon this trend, initiatives such as the European Higher Education Area (EHEA) and UNESCO’s Engineering for Sustainable Development agenda have further reinforced this transformation, emphasizing the core roles of sustainability, digital competence, and social responsibility in engineering education (Gutierrez-Bucheli et al., 2022; Chen et al., 2022). These two initiatives explicitly state that future engineers should not only possess technical abilities but also the awareness to integrate engineering practice with social responsibility, enabling them to make sustainable development decisions in an increasingly complex global environment (Kushnir et al., 2023). This policy-oriented and sustainability-driven approach not only reflects the global demand for a transformation in engineering education but also responds to the high expectations placed on engineering talent in the digital and globalized world. Engineering education must increasingly focus on interdisciplinary issues such as ecology, society, and ethics, in order to address the profound social impacts brought about by future technological transformations (Beldad and Miedema, 2025). The common feature of these frameworks and initiatives is their emphasis on the interdisciplinary nature and global vision of engineering education, pushing it to evolve from merely technical training to cultivating well-rounded, innovative talent. This transformation aligns with the industrial and societal demand for high-quality, sustainable engineers and signals that future engineering education will place greater emphasis on developing students’ ability to solve complex problems and adapt to societal needs.

However, despite the progress these frameworks have made in guiding education, engineering education still faces many challenges globally. For instance, many higher education institutions’ engineering curriculum reforms are still lagging behind, with significant gaps between the course content and the rapid pace of technological development (Xu, 2025; Ahmed et al., 2022). Furthermore, although the importance of interdisciplinary education is increasingly recognized, in practice, most courses still focus on foundational theory and computation, with insufficient attention given to fostering interdisciplinary collaboration and innovative thinking (Solodikhina and Solodikhina, 2022; Adair and Jaeger, 2016). Therefore, while international frameworks provide directional guidance for education, how to implement these philosophies in practice remains a central challenge in the current reform process (Wu et al., 2023).

### 2.2 Integration of AI in higher education

#### 2.2.1 The pedagogical role of AI

AI enables a transition from standardized instruction to personalized learning by analyzing learners’ cognitive profiles, prior performance, and behavioral data (Hariyanto et al., 2025). Through adaptive algorithms and learning analytics, AI systems can dynamically tailor instructional content, learning pace, and assessment difficulty to individual needs (Strielkowski et al., 2025; Čep et al., 2026). In this context, LLM-based systems further enhance personalization by supporting natural language interaction, formative feedback, and on-demand scaffolding aligned with learners’ conceptual understanding (Munaye et al., 2025; Abd-Alrazaq, 2023). Such personalized learning environments have been shown to enhance learner engagement, self-efficacy, and motivation, allowing students to progress along differentiated learning trajectories (Zhou et al., 2025). In higher education, AI-driven adaptive learning platforms and intelligent tutoring systems (ITS) have demonstrated particular effectiveness in STEM disciplines—such as programming, mathematics, and data science—where iterative feedback and structured problem-solving pathways can be continuously optimized (Wu et al., 2023).

(1) Personalization. AI enables a transition from standardized instruction to personalized learning by analyzing learners’ cognitive profiles, prior performance, and behavioral data (Hariyanto et al., 2025). Through adaptive algorithms and learning analytics, AI systems can dynamically tailor instructional content, learning pace, and assessment difficulty to individual needs (Plooy et al., 2024). With recent advances in generative artificial intelligence, particularly the emergence of LLM, the capacity for personalized instructional support has been further extended. LLM-based systems are able to provide natural language interaction, formative feedback, and on-demand scaffolding, thereby aligning instructional support more closely with learners’ evolving conceptual understanding (Xing et al., 2025; Peláez-Sánchez et al., 2024). Empirical studies indicate that such personalized learning environments enhance learner engagement, self-efficacy, and motivation, enabling students to progress along differentiated learning trajectories (Zhou et al., 2025).
(2) Automation. Automation refers to the application of AI technologies to streamline repetitive instructional and administrative tasks, including grading, attendance management, and feedback generation (Deepshikha, 2025). Automated essay scoring, intelligent code evaluation, and semantic-based feedback systems have significantly improved assessment efficiency and consistency while reducing instructors’ workload (Khairullah et al., 2025). Recent advances in generative AI, particularly LLMs, have further extended the scope of educational automation beyond rule-based assessment to encompass natural language understanding and generative feedback, enabling scalable formative assessment in large-enrollment courses (Xing et al., 2025). By delegating routine cognitive and administrative tasks to AI systems, educators are able to reallocate time and attention toward mentoring, critical discussion, and higher-order learning activities (Jeon and Lee, 2023). This development reflects a pedagogical shift from labor-intensive instruction toward evidence-informed and data-supported teaching models (Viberg et al., 2018).
(3) Augmentation. Rather than replacing human educators, artificial intelligence increasingly functions as an assistive intelligence that supports and enhances teaching effectiveness and pedagogical decision-making. From the perspective of instructional processes, technologies such as predictive analytics, learning dashboards, and emotion-aware systems enable the real-time analysis of student engagement, comprehension, and participation patterns, thereby providing instructors with richer and more dynamic learning insights (Cabral et al., 2025). Such data-supported insights allow teachers to move beyond intuition-based judgments and make more informed pedagogical decisions (Fairman et al., 2025). With the ongoing development of generative artificial intelligence, particularly dialogic and content-generative systems based on LLMs, the role of AI in pedagogical augmentation has been further expanded (Shi et al., 2026). These systems can assist instructors in instructional planning, question generation, learning guidance, and adaptive explanation, thereby extending teachers’ pedagogical reach while maintaining human oversight and instructional authority. This form of human–AI collaboration does not diminish the role of educators; instead, it amplifies their professional expertise and responsiveness within increasingly complex learning environments. At the instructional level, such augmentation-oriented AI tools enable instructors to identify learning bottlenecks in a timely manner, implement targeted interventions, and continuously refine instructional strategies based on empirical evidence. At the institutional level, AI-based classroom and learning analytics contribute to pedagogical governance by providing longitudinal insights into teaching quality and student performance trends, thereby supporting continuous improvement in educational design and academic management (Gašević et al., 2022).

Empirical studies indicate that AI-powered intelligent tutoring systems can improve learning efficiency, engagement, and knowledge retention (Bai et al., 2025; Lin et al., 2023). Similarly, adaptive learning platforms employ reinforcement and optimization algorithms to adjust task difficulty and provide individualized support (Tan et al., 2025). These systems have been most widely adopted in STEM domains, where large-scale datasets and well-structured knowledge representations allow machine learning models to operate with higher reliability and pedagogical effectiveness.

Taken together, personalization, automation, and augmentation should be understood as interrelated pedagogical dimensions of AI integration in higher education rather than independent technical functions. Their combined effect signals a transition from experience-driven teaching toward adaptive, dialogic, and evidence-informed pedagogical paradigms, particularly in the context of LLM-enabled learning environments. However, existing research remains largely focused on functional performance at the tool or course level, with limited attention to how AI, especially generative models, can be systematically aligned with curriculum design and long-term talent cultivation objectives. This gap highlights the need for pedagogically grounded frameworks that integrate AI capabilities with core educational values and instructional theory.

#### 2.2.2 Institutional and strategic adoption

Beyond classroom-level innovations, the integration of AI at the institutional level remains limited. Many universities adopt AI as technological augmentation rather than as part of a strategic pedagogical framework. Zawacki-Richter et al. (2019) found that only a small fraction of higher education institutions possess comprehensive AI strategies linking technology use to curriculum design, faculty training, and assessment reform. The majority of implementations occur through pilot projects or external collaborations, leading to fragmented adoption and poor scalability.

In addition, ethical concerns—such as data privacy, algorithmic bias, and transparency—pose further barriers to institutional integration (Khatri and Karki, 2023). To overcome these, scholars argue that AI in education must transition from being a technological experiment to a pedagogical ecosystem, in which AI informs instructional design, feedback loops, and institutional decision-making. This conceptual shift underpins the motivation for developing AI-driven reform frameworks in higher education (Troitskaya, 2025).

#### 2.2.3 Potential challenges and risks of AI in education

Although artificial intelligence has demonstrated significant advantages in higher education, including personalized learning, adaptive feedback, increased student engagement, and more efficient assessment, recent studies have highlighted several potential challenges and risks associated with its integration (Bittle and El-Gayar, 2025; Vázquez-Madrigal et al., 2024). A primary concern is algorithmic bias, where intelligent tutoring systems and automated grading tools may inadvertently favor certain student groups or learning styles, resulting in inequitable assessment outcomes (Baker and Hawn, 2022; Yin et al., 2025). This risk is particularly pronounced when large language models or other pre-trained AI systems reflect biases inherent in their training datasets.

Another critical issue is over-reliance on generative AI, which may reduce students’ opportunities for independent problem-solving and critical thinking (Zhai et al., 2024). Excessive dependence on AI-generated content for assignments or projects could hinder the development of deep conceptual understanding, creativity, and analytical skills, thereby potentially undermining higher education learning objectives (Zhai et al., 2024; Graham and Milan, 2025).

Beyond individual student outcomes, ethical and pedagogical considerations include the potential reduction of meaningful instructor–student interactions, diminished opportunities for professional ethics development, and unintended constraints on the breadth of interdisciplinary learning (Seo et al., 2021). These concerns underscore the importance of designing AI-integrated curricula that balance technological innovation, pedagogical oversight, institutional policy, and human-centered evaluation mechanisms (Chick, 2025).

In summary, although AI offers transformative opportunities for personalized, adaptive, and data-driven education, higher education institutions must proactively address these risks through careful design, transparent algorithmic governance, multi-dimensional evaluation, and continuous monitoring. By acknowledging both the benefits and the inherent challenges of AI integration, educators and policymakers can cultivate equitable, effective, and sustainable AI-enhanced learning environments (Alfaleh, 2026; Alfiras et al., 2025).

### 2.3 Interdisciplinary and collaborative learning in engineering education

#### 2.3.1 Theoretical foundations

Interdisciplinarity has long been recognized as a cornerstone of innovation in engineering education. Rooted in constructivist learning theory (Beldad and Miedema, 2025), it posits that knowledge construction occurs most effectively when learners engage in authentic, problem-centered experiences that transcend disciplinary silos. Kolb’s experiential learning cycle further reinforces this by linking abstract conceptualization with active experimentation—principles that underpin project-based and problem-based learning (PBL) models (Kolb, 1984; Efstratia, 2014).

In engineering contexts, interdisciplinary learning involves integrating principles from physics, mathematics, computer science, design, and management to solve complex societal or industrial problems. It fosters systems thinking, cognitive flexibility, and reflective practice, all of which are essential for navigating uncertainty in professional practice. Collaborative approaches, such as challenge-based learning (CBL) and transdisciplinary design studios, have proven effective in developing these competencies (Doulougeri et al., 2024; Taconis and Bekker, 2023).

#### 2.3.2 The “new engineering education” movement

In China, the New Engineering Education (NEE) initiative, launched by the Ministry of Education in 2017, represents a national effort to modernize engineering training for the AI era. NEE emphasizes outcomes-based education (OBE), industry engagement, and scenario-driven learning (Zhuang and Xu, 2018; Wei and Zhang, 2025). It encourages universities to collaborate with enterprises in curriculum co-design, create interdisciplinary research platforms, and align teaching objectives with industrial innovation needs (Zheng and Lin, 2021; Shen and Li, 2020).

Empirical research on NEE has documented improvements in students’ practical abilities, innovation literacy, and interdisciplinary awareness (Li, 2020; Han and Liu, 2018). However, implementation varies widely among institutions. Many universities still face obstacles such as insufficient faculty expertise in AI and data analytics, rigid credit structures, and limited incentives for interdisciplinary teaching (Mah and Groß, 2024; Bednarowska-Michaiel and Uprichard, 2025). Consequently, NEE’s full potential remains unrealized without stronger theoretical grounding and technological integration.

### 2.4 AI–interdisciplinary integration: current Progress and gaps

Recent literature has begun to explore the intersection between AI integration and interdisciplinary learning, recognizing that both share the goal of preparing learners for complexity, collaboration, and continuous innovation. AI can serve as both content (a knowledge domain to be mastered) and methodology (a tool for enhancing learning across domains; Cai et al., 2025; Yan and Qianjun, 2025). For example, in computational biology, AI-driven design, and smart manufacturing, students apply machine learning techniques to model biological systems or optimize production processes—illustrating how disciplinary boundaries can be bridged through AI-enabled cognition.

However, despite such progress, research remains fragmented across separate scholarly communities—educational technologists study AI pedagogy, while engineering educators focus on interdisciplinary curriculum design (Schleiss et al., 2024). There is a scarcity of studies offering integrated theoretical frameworks that explain how AI can structurally support interdisciplinary learning environments. In addition, few models exist for embedding AI within the entire talent cultivation process, from curriculum planning and teaching delivery to assessment and professional practice.

Three major gaps can be identified in the current literature:

Fragmentation of Application: AI tools are often confined to individual courses or short-term experiments rather than systematically embedded in curricula.
Lack of Institutional Frameworks: Most universities lack governance models linking AI adoption with pedagogical outcomes and quality assurance mechanisms.
Weak Industry Linkages: Industrial partners are rarely engaged in co-developing AI-driven interdisciplinary learning ecosystems, limiting authenticity and relevance.

These gaps underline the necessity of a comprehensive reform model that unites AI, interdisciplinarity, and industry collaboration under a single pedagogical philosophy.

Building on the identified gaps, this study proposes a comprehensive AI-driven educational reform framework that directly addresses these shortcomings while providing measurable research objectives. Specifically, to mitigate the fragmentation of AI tool applications, AI technologies are systematically integrated across curriculum structures, instructional processes, and practice-oriented modules (Sections 3.1 and 3.2), ensuring their consistent application throughout the educational process and enhancing students’ technical proficiency and practical competencies. To establish an institutionalized framework for reform, a multi-dimensional evaluation system and intelligent teaching ecosystem have been implemented (Section 3.5), employing standardized assessment criteria and data-driven feedback mechanisms to support curriculum optimization, instructional design, and the systematic development of student capabilities. Finally, to strengthen industry engagement and address the weak linkage between academic training and professional practice, the framework incorporates interdisciplinary course design, practice-based projects, enterprise-affiliated laboratories, and a dual-mentor system (Sections 3.3 and 3.4), effectively integrating industry resources into educational practice and fostering students’ interdisciplinary collaboration, innovation capacity, and preparedness for real-world challenges. By systematically aligning these measures with the identified research gaps, the framework provides a coherent and testable model for AI-driven talent cultivation in computer-related disciplines.

## 3 Reform framework and implementation

To address the disconnect between traditional talent cultivation models and the demands of emerging industries under the paradigm of new quality productive forces, this study proposes a comprehensive and multi-dimensional reform framework for computer-related majors. The framework is structured around five core components: curriculum restructuring, smart teaching integration, interdisciplinary and collaborative talent cultivation, practice-based learning enhancement, and multi-dimensional evaluation. These components form an interconnected system designed to deliver future-oriented, AI-empowered talent cultivation with both academic depth and industrial relevance.

### 3.1 AI-driven curriculum restructuring

Curriculum restructuring forms the foundation of this initiative, designed to bridge the gap between academic knowledge and emerging industrial needs under the paradigm of AI-driven productive forces (Padovano and Cardamone, 2024). Rather than pursuing isolated course updates, the reform establishes an integrated, competency-oriented curriculum system that aligns disciplinary content with frontier technologies and the evolving skill demands of the AI-driven era (Zhong et al., 2025).

(1) Framework Design and Competency Mapping.

**Figure 1:**
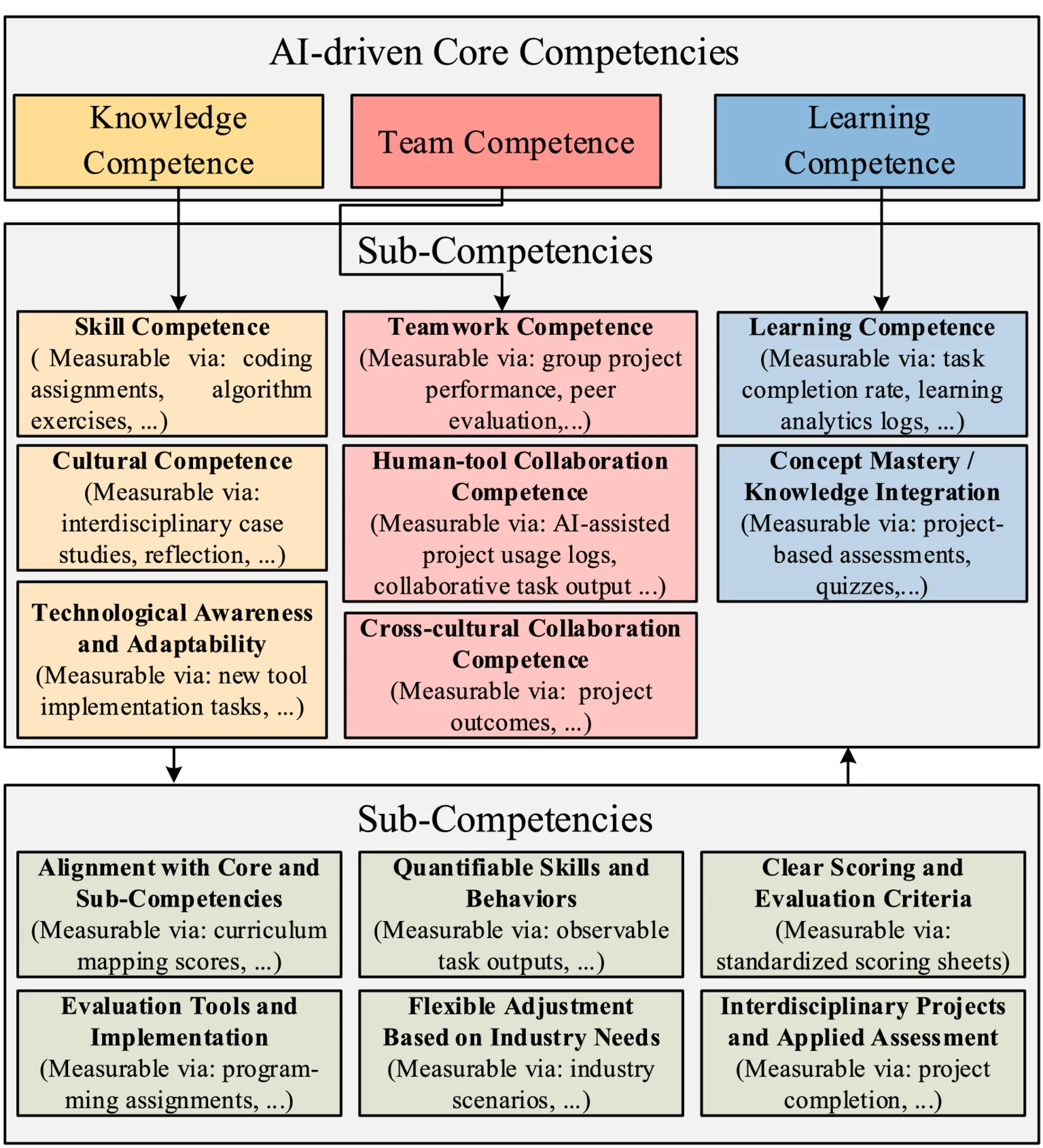
Competency map framework: Linking graduate attributes to observable skills and measurable behaviors.

To operationalize these competency goals, a competency map was developed to translate abstract professional requirements into measurable learning outcomes. As shown in Figure 1, this map is structured in three hierarchical layers that link high-level graduate attributes to observable skills and measurable behaviors:

(1) AI-driven Core Competencies, representing the overarching graduate attributes required by industry;
(2) Sub-competencies, which specify observable skills such as algorithmic reasoning, model construction, data visualization, and secure system design;
(3) Performance indicators, defining quantifiable behaviors and outputs used for assessment.

In the design of the AI-driven Core Competencies, the competency framework proposed by Huang (Huang, 2021) was employed, as it has gained widespread recognition and application in the field of artificial intelligence education, particularly within China’s educational reforms. These competencies form the foundation of the curriculum framework and are essential for preparing students to thrive in the AI-driven era. The following sections will delve into each of these competencies, outlining their significance and how they can be cultivated through AI-focused education.

(1) Knowledge Competence encompasses not only the foundational technical skills in artificial intelligence and computer science—such as programming, algorithms, machine learning, and data analysis—but also an understanding of the social, cultural, and ethical implications of these technologies. This dual focus on technical proficiency and cultural awareness is crucial for developing students’ ability to apply AI in a responsible and socially aware manner.
(2) Team Competence refers to students’ ability to collaborate effectively in team settings, emphasizing communication, coordination, and collective problem-solving. In the context of AI education, it also highlights the integration of AI systems and tools within team projects, teaching students to leverage these technologies to enhance team productivity and innovation.
(3) Learning Competence includes both cognitive and self-directed learning abilities, requiring students to be capable of analyzing, reasoning, and solving complex problems while also fostering the ability to engage in lifelong learning. Given the rapid advancements in AI, students must continuously update their skills and knowledge through independent study. Through the integration of these competencies in the curriculum, students are better prepared to navigate the challenges of the AI-driven era and contribute to technological innovation.

In addition to the six sub-competencies proposed by Huang, we further expanded upon this framework by incorporating two additional sub-competencies: Technological Awareness and Adaptability Competence and Cross-cultural Collaboration Competence. This expansion reflects the evolving nature of AI-driven education and the increasingly globalized, technology-centric world in which students must develop their skills. The decision to expand Huang’s original framework was based on the recognition that the fast-paced developments in artificial intelligence and the broader technological landscape demand that students possess not only technical expertise but also the ability to quickly adapt to new tools and technologies. Technological Awareness and Adaptability Competence emphasizes students’ ability to stay informed about emerging technologies and their capacity to adapt to new, unforeseen technological changes. This sub-competency is essential as AI technologies continue to evolve at an unprecedented rate, requiring students to be flexible and proactive in acquiring new skills. Furthermore, in an interconnected world, the ability to collaborate across cultural and geographic boundaries has become more crucial than ever. Cross-cultural Collaboration Competence aims to equip students with the necessary skills to effectively communicate, collaborate, and problem-solve in diverse, multicultural teams. As AI technologies are developed and deployed globally, understanding different perspectives and working seamlessly with individuals from various cultural backgrounds will be key to driving innovation and success in the AI-driven era.

In the AI-driven curriculum framework, Performance Indicators (PIs) serve as critical tools for translating abstract learning goals into measurable and actionable assessment standards (Junaidi et al., 2025). To ensure the effectiveness of the PIs, we have adopted the following approach:

(1) Alignment with Core and Sub-Competencies. The design of Performance Indicators ensures a direct alignment with the Core Competencies (such as Knowledge Competence, Team Competence, and Learning Competence) and their associated sub-competencies (such as Skill Competence, Cultural Competence, and Teamwork Competence; Vargas et al., 2025; Ponomariovienė et al., 2025). For instance, for Knowledge Competence, the Performance Indicators may include “the ability to solve practical machine learning problems through programming and algorithms” or “the ability to analyze and interpret the outputs of AI models.” These behaviors directly assess students’ grasp of foundational knowledge and their application skills.
(2) Quantifiable Skills and Behaviors. Each Performance Indicator is defined as a specific, observable action. For example, “students are able to use a specific machine learning algorithm (e.g., linear regression, neural networks) to process a dataset and produce actionable results.” This behavior can be measured through students’ actual performance, with clear outputs (such as code or model accuracy) forming the quantifiable standards.
(3) Clear Scoring and Evaluation Criteria. To ensure the feasibility of these Performance Indicators, each one is accompanied by explicit scoring rubrics. For example, for Team Competence, specific criteria might be set such as: i) Students must effectively communicate and propose at least three different solutions during team discussions and participate in decision-making processes; ii) Contributions are assessed based on both the individual student’s input (e.g., proposed solutions, roles in the team) and the project deliverables (e.g., quality of final results). These behaviors serve as the basis for quantifying the student’s teamwork performance.
(4) Evaluation Tools and Implementation. Every Performance Indicator is paired with assessment tools, such as programming assignments, project reports, and peer reviews. For example, for the indicator “apply machine learning models to solve practical problems,” students’ code quality, model accuracy, experimental design, and problem-solving capacity are quantified, with scores assigned (e.g., a 10-point scale where code quality accounts for 4 points, model accuracy for 3 points, and problem-solving for 3 points).
(5) Flexible Adjustment Based on Industry Needs. To ensure the continued relevance and applicability of the Performance Indicators, these standards will be reviewed and adjusted regularly (e.g., every semester). Feedback from students, as well as input from industry experts and academic faculty, will be used to revise and optimize these evaluation criteria. This process ensures that the Performance Indicators remain up-to-date with technological developments and industry requirements. For instance, if a particular machine learning technique or tool becomes more widely used, the associated performance indicator and evaluation criteria may be updated accordingly.
(6) Interdisciplinary Projects and Applied Assessment. The Performance Indicators are not limited to theoretical knowledge but also include practical testing through interdisciplinary projects (Zhao et al., 2023; Wu et al., 2024). For example, students must integrate AI technologies with knowledge from other fields (such as mechanical engineering or chemical engineering) to solve real-world, multi-faceted problems. The Performance Indicators for these projects could include: “students must successfully apply AI technologies within interdisciplinary teams and work collaboratively with domain-specific experts to solve integrated technical challenges.”

(2) Interdisciplinary Collaboration and Knowledge Integration.

In the process of AI-driven curriculum restructuring, interdisciplinary collaboration and knowledge integration have become central components (Cai et al., 2025). These are designed to ensure that students not only master AI technologies but also apply them to solve real-world complex problems (Southworth et al., 2023). To achieve both academic rigor and industrial relevance, the university has formed close collaborations with leading enterprises and research institutes, establishing interdisciplinary curriculum development teams. These teams are dedicated to integrating cutting-edge AI technologies with traditional disciplinary knowledge, fostering innovation and development in the curriculum, and ensuring that the courses meet the future needs of students in their careers and research.

By modularizing frontier knowledge areas such as artificial intelligence, industrial internet security, and big data technologies, and systematically embedding them across foundational and advanced professional courses, the curriculum not only expands students’ technological horizons but also promotes the deep integration of AI with other disciplines. These modules include both theoretical AI concepts and practical skills needed for applying AI in real-world scenarios, ensuring that students are equipped to address the emerging challenges brought about by the AI-driven era.

Furthermore, the curriculum integrates knowledge from fields such as chemical engineering, mechanical engineering, and control systems, providing students with interdisciplinary learning resources. Leveraging knowledge graphs and virtual simulation technologies, the curriculum creates immersive, intelligent learning experiences, allowing students to practice and solve real-world problems within simulated environments. This approach enhances students’ theoretical foundations while also fostering practical understanding of AI technologies, enabling them to integrate AI with knowledge from other domains effectively.

Through interdisciplinary learning communities, students engage in project-based learning, collaborating with peers from diverse disciplines to solve complex, multifaceted problems. These communities not only foster resource sharing and interdisciplinary communication but also enhance students’ teamwork abilities and innovative thinking across disciplines. The core goal of these learning communities is to develop students’ capacity to solve complex problems in the AI-driven era, while strengthening their ability to collaborate across disciplines and drive innovation.

In the design of the curriculum framework, interdisciplinary collaboration and knowledge integration are directly linked to the AI-driven core competencies, ensuring that students not only gain deep knowledge of AI technologies but also apply them in interdisciplinary teams to solve complex problems. This integration equips students to thrive in a rapidly changing technological environment, preparing them to become globally-minded, cross-disciplinary innovators who can contribute to the advancement of AI in diverse sectors. Through this curriculum restructuring, students will be well-prepared to tackle the challenges of an AI-driven world and contribute to the technological and social transformation led by AI technologies.

(3) AI-Driven Curriculum Reform Structure.

**Figure 2:**
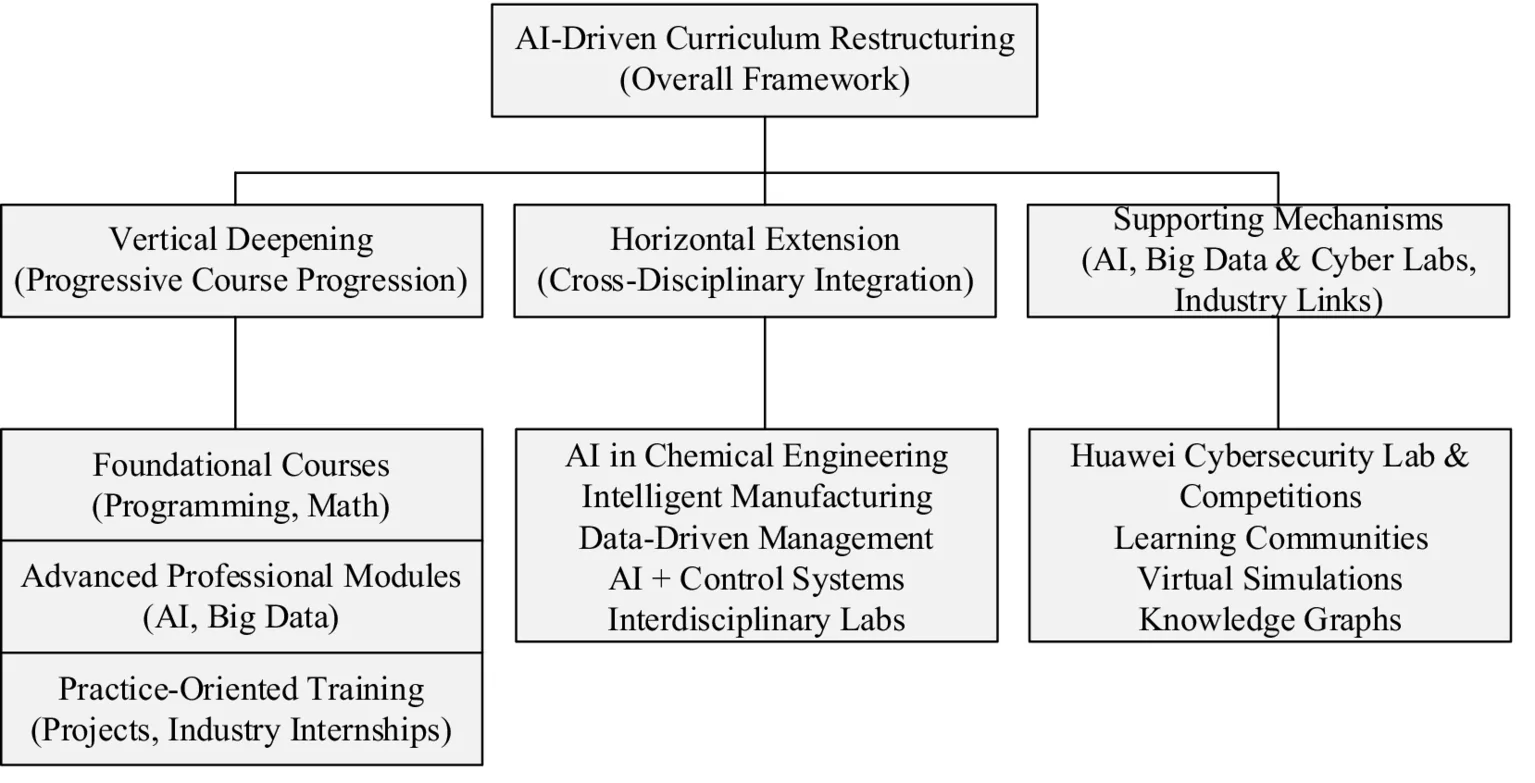
AI-Driven Curriculum Reform Framework.

To visualize the overall structure of this reform, Figure 2 presents the AI-driven curriculum reform framework.

1) The diagram illustrates how vertical progression and horizontal integration together shape an interconnected, multi-layered curriculum system.
2) The vertical dimension emphasizes progressive deepening, moving from foundational computing courses to advanced professional modules and practice-oriented training. This ensures the stepwise cultivation of students’ computational thinking and engineering competencies while consolidating their theoretical foundation.
3) The horizontal dimension represents cross-disciplinary integration, embedding AI technologies into domains such as chemical engineering, intelligent manufacturing, and data-driven management

This enables the blending of computational methods with domain-specific expertise and fosters interdisciplinary innovation. Furthermore, the curriculum strengthens AI education through new AI-related courses and hands-on project practices that engage students in real-world AI development. It expands big-data skill training via specialized laboratories and interdisciplinary competitions, and enhances cybersecurity competence through courses on network security, offensive–defensive techniques, and information security, supported by partnerships such as the Huawei Cybersecurity Laboratory.^1^ Students also participate in national cybersecurity competitions and simulated real-world attack–defense scenarios, further consolidating their applied technical skills.

As illustrated in Table 1, the integration of AI into the curriculum is demonstrated through a selection of representative courses that highlight the breadth of the reform. These include newly introduced courses such as Artificial Intelligence and Big Data Analysis, Python Programming, and Mobile Internet Technology, as well as the redesigned traditional compulsory courses like Network Security Technology and Overview of Internet of Things Technology. Additionally, practice-oriented modules like Enterprise Software Development Practice and Analysis of Network Security Protocol showcase the hands-on, application-driven nature of the reform. By embedding AI tools, industry datasets, and case studies into both theoretical and practical courses, the curriculum ensures that students not only acquire solid foundational knowledge but also gain practical experience in applying AI to real-world, domain-specific challenges.

**Table 1:** Curriculum restructuring through AI-enhanced course design.

Course category	Course name	AI integration highlights
Compulsory	Network Security Technology	Introduces AI-driven intrusion detection systems; students apply machine learning models to identify abnormal network traffic.
	Network Programming Technology	Incorporates intelligent code analysis tools for real-time debugging and performance optimization.
	Overview of Internet of Things Technology	Applies AI-based predictive models and edge computing algorithms for IoT data flow optimization.
	Network Engineering Technology Competition	Embeds AI case studies into competition tasks, such as intelligent routing and automated anomaly detection.
Elective	Artificial Intelligence and Big Data Analysis	Core elective introducing Python, TensorFlow, and big data frameworks; students complete AI-driven data analysis projects.
	Advanced Network Programming Technology	Employs AI-assisted testing platforms to evaluate large-scale programming assignments.
	Python Language	Foundation for AI practice; prepares students for deep learning and data analysis courses.
	Mobile Internet Technology	Integrates mobile AI applications such as intelligent recommendation systems and natural language interfaces.
Practice	Enterprise Software Development Practice	Uses AI-powered automated testing and code quality assessment tools to enhance project reliability.
	Analysis of Network Security Protocol	Applies NLP-based protocol parsing to identify anomalies in communication flows.
	Comprehensive Practice of Network Security Planning and Implementation	Students use AI-based simulation tools to design and evaluate security strategies.
	Planning and Practice of IoT Management Platform	Incorporates AI-enabled monitoring and prediction functions in IoT platforms.
	Network Test and Evaluation	Employs AI-driven performance evaluation systems for network benchmarking.

### 3.2 Smart teaching and learning integration

The integration of AI technologies into the teaching process represents a transformative shift in instructional design and classroom dynamics. Rather than functioning as isolated digital tools, AI systems now operate as an intelligent instructional ecosystem that connects students, instructors, and learning analytics in real time (Rojas and Chiappe, 2024). This transformation redefines how learning content is delivered, how student engagement is monitored, and how instructional decisions are made (Hariyanto et al., 2025; Sajja et al., 2025). AI-driven teaching follows the principles of data-informed pedagogy and adaptive learning theory, emphasizing continuous diagnosis, personalized intervention, and evidence-based feedback (Tan et al., 2025). By leveraging large-scale learning data, AI technologies enable instructors to understand learners’ cognitive states, predict learning bottlenecks, and tailor instruction accordingly. This shift moves the traditional model of teaching “by experience” toward one driven by quantitative insight and dynamic adaptability.

Before instruction begins, AI-powered diagnostic systems analyze historical learning records, course performance data, and individual cognitive profiles to assess knowledge readiness. The system then automatically generates personalized pre-class preparation materials, including targeted review exercises addressing individual weaknesses, visualized concept maps illustrating key topic relationships, and short AI-generated tutorial videos for self-paced microlearning. These materials enable students to enter class sessions with differentiated prior knowledge, thereby increasing teaching efficiency and supporting flipped classroom pedagogy. Instructors, in turn, receive dashboard-based overviews that highlight learning gaps across the class cohort, allowing them to customize lesson emphasis and allocate time more strategically.

During class, AI-enabled smart assistants embedded in the classroom environment monitor engagement and interaction in real time through natural language processing, gesture recognition, and participation analytics (Sajja et al., 2024). When the system detects a drop in attention or comprehension, it can push supplementary multimedia explanations, trigger real-time mini quizzes, or send alerts to the instructor for timely pedagogical intervention. This adaptive mechanism ensures that instruction remains responsive to real-time learning dynamics, preventing students from falling behind and maintaining an equitable learning pace. Instructors are also supported by automated note summarization, speech-to-text transcription, and emotion recognition functions that enhance awareness of class sentiment and learning mood, helping them adapt their teaching rhythm to classroom dynamics.

After class, AI tools continue to function as analytical and evaluative engines. Intelligent grading systems automatically assess assignments, coding projects, and even open-ended responses using semantic similarity algorithms and rubric-based evaluation models. Students receive immediate formative feedback, including error explanations and recommended remedial resources, while instructors access learning analytics dashboards that visualize trends in performance, participation, and concept mastery. These data are further fed back into the institutional learning management system (LMS), forming a closed-loop mechanism that connects learning design, implementation, and continuous improvement. Over multiple semesters, longitudinal analysis of student progress and instructional effectiveness provides evidence for curriculum optimization and teacher professional development.

The AI systems employed in this study play a critical role throughout the teaching process. The intelligent dashboard was implemented in a Python 3.10 environment using Dash and Plotly for visualization, and integrated with a PostgreSQL database to store students’ activity logs and analysis results. The adaptive feedback loops were implemented using a reinforcement learning model in PyTorch, dynamically adjusting learning tasks and personalized exercises to optimize students’ learning trajectories. For semantic similarity analysis and evaluation of open-ended assignments and project reports, texts were first converted into vector representations using the Qwen-7B pre-trained large language model, followed by computation of cosine similarity between student submissions and reference documents or existing works. This combination of technologies supports formative feedback, enables data-driven instructional interventions, ensures academic integrity, and fosters authentic student innovation.

The integration of AI into education also redefines the teacher’s role. Instead of acting solely as knowledge transmitters, instructors become learning designers and facilitators. AI handles repetitive cognitive tasks—grading, diagnostics, and data tracking—while human educators focus on mentoring, critical discussion, and emotional support. Through this human–AI collaboration, teaching becomes more student-centered, diagnostic, and reflective, aligning with the broader vision of cultivating innovative, autonomous, and adaptive learners in the AI-driven era. At the institutional level, smart teaching is supported by an integrated AI–LMS platform that connects data from classrooms, laboratories, and online learning environments. Real-time analytics inform course scheduling, content revision, and performance evaluation, enabling evidence-based decision-making. Pilot studies within computing-related majors have demonstrated measurable improvements in student engagement, self-efficacy, and learning outcomes. Teachers reported reduced administrative workload and enhanced insight into learner diversity. More importantly, the AI-driven teaching ecosystem has established a sustainable feedback mechanism that promotes continuous educational improvement, bridging the traditional divide between teaching and research in higher education.

### 3.3 Interdisciplinary and collaborative talent cultivation

In response to the growing demand for cross-disciplinary innovation, the reform initiative places strong emphasis on breaking down disciplinary silos and fostering collaborative talent cultivation. A cross-disciplinary curriculum alliance has been established, involving faculties from computer science, engineering, automation, data science, and business (Akhtar et al., 2024). Through this alliance, new courses such as “AI and Intelligent Manufacturing,” “Data Science for Industrial Process Optimization,” and “Digital Economy and Smart Governance” have been developed to integrate knowledge from multiple domains. These courses are designed to help students synthesize diverse perspectives and apply computational thinking to solve real-world, multifaceted problems.

Beyond curriculum integration, the reform also establishes a dual-mentor system that connects students with both academic advisors and industry mentors. This structure enables students to participate in real-world projects sourced directly from enterprise partners. Under joint supervision, students work on problems such as cybersecurity simulations, big data analytics for business intelligence, and AI-based fault detection systems in manufacturing environments. These projects not only bridge theory and practice but also cultivate students’ communication, coordination, and project management skills.

Moreover, the reform encourages active cooperation between institutions. Joint teaching arrangements, shared elective platforms, and collaborative innovation competitions have been implemented in partnership with peer universities. For instance, students from multiple institutions co-enroll in specialized online courses and participate in cross-campus graduation projects. This inter-institutional collaboration facilitates resource sharing, expands exposure to different academic cultures, and promotes a vibrant innovation ecosystem beyond the boundaries of a single institution.

Collaboration with leading enterprises such as Huawei and Neusoft has been particularly instrumental in enhancing the reform’s practical orientation. These partnerships have led to the co-construction of R&D laboratories, shared training platforms, and industry-certified learning modules. Enterprise professionals are regularly invited to deliver guest lectures, evaluate student projects, and offer feedback on curriculum design. Through these collaborative mechanisms, students gain direct access to cutting-edge industrial technologies, workplace practices, and entrepreneurial thinking, all of which significantly enrich their educational experience and career preparedness.

### 3.4 Practice-oriented learning platforms

Recognizing that innovation is rooted in practical experience, the reform emphasizes a practice-centered learning paradigm that closely integrates real-world industrial contexts with advanced educational infrastructure (Lavado-Anguera et al., 2024). This approach is guided by the principle that knowledge acquisition and innovation capability are mutually reinforced through continuous cycles of experimentation, reflection, and application (Liu and Li, 2025). To this end, a multi-tiered system of practice environments has been established, combining physical laboratories, virtual platforms, and enterprise-linked experiential programs to provide students with a holistic and authentic learning experience.

At the physical level, the college has invested in the construction of several specialized facilities, including an AI Simulation Laboratory, a Big Data Experimental Center, and a Cybersecurity Sandbox. These environments are equipped with professional-grade servers, GPU computing clusters, and enterprise-level software suites such as TensorFlow, PyTorch, Hadoop, and Wireshark. Students conduct machine learning experiments, analyze large-scale datasets, and perform network intrusion simulations that mirror real-world industry practices. The AI Simulation Laboratory supports model training on real-time data streams collected from partner enterprises, enabling students to apply theoretical algorithms to real industrial problems such as fault prediction, image recognition, and intelligent decision optimization. The Big Data Experimental Center facilitates data preprocessing, storage, and analytics under authentic industrial data pipelines, while the Cybersecurity Sandbox provides isolated environments for offensive and defensive exercises, penetration testing, and cryptography practice—ensuring that students gain technical mastery in a secure, realistic, and ethically guided setting.

In parallel with these physical facilities, the reform has developed a comprehensive suite of virtual learning platforms designed to transcend spatial and temporal limitations. These include cloud-based programming environments, virtual factories for process simulation, and AI-driven online collaborative coding spaces. Through these digital ecosystems, students can seamlessly access programming tasks, simulate industrial workflows, and collaborate in real-time with peers from different disciplines or institutions. Virtual hackathons and cloud-based design competitions are regularly hosted, encouraging students to engage in peer-to-peer learning and problem-solving across geographic and disciplinary boundaries. These platforms are fully integrated with the smart teaching ecosystem described in Section 3.2, enabling dynamic synchronization between theoretical instruction, experimental training, and formative assessment.

To further strengthen experiential learning and innovation-driven education, the college organizes a broad portfolio of AI + Industry innovation challenges, algorithm design contests, and interdisciplinary entrepreneurship incubators. Each of these initiatives is aligned with course outcomes and academic credit systems, ensuring that participation in innovation activities directly contributes to formal learning progress. Students are encouraged to propose AI-based solutions to real industrial challenges, such as predictive maintenance for intelligent manufacturing, energy consumption optimization, and intelligent logistics scheduling. Outstanding projects are showcased at annual Innovation and Entrepreneurship Festivals and recommended for participation in national-level competitions such as the “Internet+ Innovation Challenge” and the “Blue Bridge Cup.” This dual recognition—academic and professional—provides external validation for student achievements and deepens their motivation for lifelong learning and innovation.

In addition to course-based practice, a data-driven internship and project management platform powered by AI analytics has been implemented to monitor students’ off-campus engagement. The system captures daily work logs, reflective journals, and feedback from enterprise mentors, which are automatically analyzed to generate visualized progress dashboards and performance analytics. Supervising instructors can thus track competency development in real-time, provide timely guidance, and align internship tasks with learning outcomes. Enterprise mentors, on the other hand, can input structured evaluations focusing on creativity, collaboration, and problem-solving skills. This feedback loop between academia and industry transforms internships from mere observational experiences into evidence-based learning processes.

Furthermore, international collaboration mechanisms have been introduced to expand the scope of practice learning. Joint laboratories with global partners—such as research institutes in Singapore, Germany, and the United States—facilitate student participation in cross-border AI innovation projects. Through online co-supervision and international summer research programs, students experience diverse technological ecosystems and develop intercultural collaboration skills essential for global competitiveness.

The practice-oriented framework also emphasizes the cultivation of soft skills and professional literacy, including communication, teamwork, project management, and ethical responsibility in AI application. Reflection-based assessment encourages students to evaluate their own learning trajectories, identify weaknesses, and set personal development goals. Continuous feedback from mentors and peers fosters a growth mindset and resilience in problem-solving.

Overall, the establishment of this practice ecosystem marks a critical transformation from knowledge transmission to experiential, competency-based learning. It ensures that students are not only proficient in coding or data modeling but also capable of integrating theory with practice, innovating across disciplinary boundaries, and adapting to fast-evolving technological contexts. The combination of physical laboratories, virtual environments, enterprise engagement, and international collaboration collectively builds a robust platform for cultivating high-level computing professionals equipped with both technical excellence and adaptive intelligence—traits essential for leadership in the era of AI-driven productive forces.

### 3.5 Multi-dimensional evaluation system

A robust and practical multi-dimensional evaluation system has been established to assess student outcomes holistically across cognitive, practical, innovative, and professional dimensions. Unlike traditional exam-centered models that focus primarily on knowledge recall, this framework integrates performance-based assessments, behavioral analytics, and authentic application evaluations, forming a continuous feedback loop that connects learning, teaching, and institutional improvement. Grounded in the principle of constructive alignment, this system ensures that each assessment element is directly aligned with learning objectives, instructional methods, and competency standards. Its goal is not only to measure learning outcomes but also to promote learning as an ongoing, evidence-driven process that evolves through data, feedback, and reflection.

In the cognitive dimension, students’ mastery of computing fundamentals, algorithmic reasoning, and artificial intelligence principles is evaluated using a blend of formative and summative assessments. Formative assessments include online quizzes, automated coding exercises, and adaptive problem sets generated by the AI-based learning platform, providing real-time insights into students’ conceptual understanding and learning behaviors. Summative assessments focus on higher-order skills such as system design, data modeling, and algorithm optimization, assessed through project presentations and code walkthroughs. The AI-driven learning analytics platform continuously collects data such as task completion rates, accuracy, and time-on-task, transforming these into visualized learning trajectories that help instructors identify knowledge gaps, adjust instruction, and support differentiated teaching strategies.

Innovation capability is another critical dimension of evaluation. Students are assessed not only on the accuracy or efficiency of their solutions but also on their originality, feasibility, and practical impact. Their participation in competitions, innovation labs, and research projects is recorded and analyzed, with tangible outputs—such as patents, published papers, or prototype demonstrations—incorporated into innovation performance metrics. To ensure fairness and transparency, rubrics have been established to assess creative thinking, interdisciplinary integration, and technical execution. The college’s AI platform also employs semantic similarity analysis to evaluate the novelty of project reports and research proposals, helping to ensure academic integrity and foster authentic innovation. Specifically, the text is first transformed into vector representations using the Qwen-7B pre-trained large language model, and then cosine similarity is calculated between student submissions and reference documents or existing works. This approach not only helps ensure academic integrity but also promotes authentic innovation through quantitative novelty assessment. The analysis was implemented in a Python 3.10 environment using the HuggingFace Transformers framework and integrated with a PostgreSQL database to store student submissions and analysis results.

In evaluating professional competence and soft skills, the system combines both quantitative and qualitative data. Peer reviews, enterprise mentor feedback, and reflective writing assignments provide multidimensional perspectives on communication, collaboration, leadership, and ethical responsibility. During internships and enterprise-linked projects, mentors provide structured digital evaluations that focus on adaptability, teamwork, and professionalism in real-world industrial settings. Reflective reports written by students are analyzed using natural language processing algorithms to assess the depth of reflection, logical coherence, and ethical awareness. This hybrid evaluation system—combining human judgment with AI analytics—ensures that both cognitive and affective dimensions of learning are captured with precision and fairness.

All assessment data converge into an AI-powered institutional dashboard, which aggregates and visualizes student performance at both individual and cohort levels. The dashboard synthesizes data on cognitive progress, innovation outputs, engagement levels, and professional skill development, offering educators a comprehensive view of student growth. Instructors can use this information to refine course design, identify struggling students, and provide personalized feedback, while program coordinators analyze aggregated data to guide curriculum adjustments and faculty development planning. At the institutional level, longitudinal analytics are employed to assess program outcomes, detect emerging trends, and guide strategic educational reforms. Thus, evaluation becomes a dynamic driver of continuous improvement, rather than a static endpoint.

To ensure the smooth operation of this data-driven evaluation system, the college has implemented a comprehensive faculty development program focused on AI-assisted pedagogy and learning analytics. Instructors undergo certification training on platforms such as Rain Classroom and Learning Pass, participate in workshops on data-informed assessment design, and engage in cross-departmental mentorship programs to promote pedagogical innovation. These initiatives enhance educators’ ability to critically interpret AI-generated insights and apply them ethically in instructional decision-making. Furthermore, an ethical governance framework has been introduced to ensure fairness, accountability, and transparency in algorithmic grading. Teachers retain oversight authority for all AI evaluations, and audit trails are maintained to detect bias or inconsistencies in automated scoring.

Over time, the implementation of this multi-dimensional evaluation system has led to measurable improvements in both teaching quality and student outcomes. Students show stronger self-regulation, greater engagement in project-based learning, and better integration of theory and practice. Faculty members report reduced grading workloads, improved diagnostic accuracy, and increased capacity for reflective teaching. Program-level analyses reveal positive correlations between the adoption of AI-enabled feedback mechanisms and gains in innovation output, collaboration, and academic performance. Ultimately, this system serves as an engine for sustainable educational transformation, aligning learning outcomes with industrial demands, enabling adaptive teaching through real-time data, and fostering a culture of continuous improvement that defines the essence of AI-driven education.

## 4 Results and evaluation

Since the implementation of the reform initiative, the proposed AI-empowered talent cultivation model has undergone phased deployment across four undergraduate programs: Computer Science and Technology, Software Engineering, Network Engineering, and Data Science and Big Data Technology. A total of 244 students participated in the study, and data were collected over two consecutive semesters. Instruction and supervision were provided by five faculty members, who were actively involved in both teaching and assessment activities throughout the implementation period. This comprehensive involvement ensured consistency in instructional delivery and evaluation across all participating courses and cohorts. The evaluation of the outcomes focuses on both quantitative indicators and qualitative feedback to assess the effectiveness of the reform in improving student learning, teaching quality, and industry alignment.

### 4.1 Improvement in student competency and engagement

To evaluate the effectiveness of the AI-driven educational reform, this study conducted a comparative analysis using student performance data from two consecutive academic years. Baseline data were collected from the cohort of students who completed the course prior to the implementation of the reform, whereas post-reform data were obtained from the subsequent cohort that completed the same course following the introduction of the AI-enhanced curriculum. The comparative data were drawn from students of the same computer-related majors but from different academic years. The analysis focused on the following metrics:

(1) Programming Project Completion Rate: The proportion of assigned programming tasks completed by each student, serving as an indicator of practical competency and task fulfillment.
(2) Algorithmic Reasoning Assessment: Student performance on programming assignments and algorithmic problem sets was evaluated to measure logical reasoning, problem analysis, and solution development capabilities. Scoring was conducted by instructors based on code correctness, logical rigor, algorithmic efficiency, and the appropriateness of problem-solving approaches.
(3) Teamwork and Project Management Competence: Assessed through group project outputs and course reports, with instructors evaluating communication, task allocation, and the quality of project deliverables.

Both cohorts completed the tasks under identical assessment criteria to ensure comparability. As the reform was implemented program-wide, a parallel control group was not available; therefore, the analysis is based on a descriptive comparison between cohorts. To ensure fairness, the evaluation of programming project completion, algorithmic reasoning, and teamwork and project management was conducted using consistent methods aligned with the pre-reform assessment standards, since pre-reform data could not be automatically scored.

Preliminary results indicate that students in the post-reform cohort demonstrated substantial improvements across multiple competency dimensions (Table 2). Specifically, compared to the pre-reform cohort, the average programming project completion rate increased by 22.5 percent, and the mean algorithmic reasoning assessment score increased by 18 percent. Moreover, students displayed stronger teamwork and project management capabilities, as reflected in peer evaluation results and group project outcomes. Compared with the pre-reform cohort, the post-reform cohort exhibited significant improvements across all measured competency domains. Specifically, the average programming project completion rate increased by 22.5%, the mean algorithmic reasoning score increased by 18.2%, and the teamwork and project management score increased by 19.4%. Independent-samples t-tests confirmed the statistical significance of these differences (*p* = 4.897*10^−17^, 2.668*10^−12^, and 3.397*10^−12^, respectively), indicating that the observed enhancements are highly unlikely to have occurred by chance. The 95% confidence intervals for the mean differences further support the robustness of these improvements: 13.48–16.57 for programming project completion rate, 11.23–14.23 for algorithmic reasoning score, and 1.05–1.63 for teamwork and project management score, demonstrating that the observed gains are both statistically and practically meaningful. Moreover, the corresponding Cohens d values (0.83, 0.68, and 0.67, respectively) indicate moderate to large practical effects, further confirming the substantive impact of the reform on students’ technical proficiency, algorithmic reasoning, and collaborative capabilities.

**Table 2:** Comparison of student competencies before and after reform.

Evaluation metric	Pre-reform cohort average	Post-reform cohort average	Improvement
Programming project completion rate (%)	72.3	88.6	+22.5%
Algorithmic reasoning assessment score (out of 100)	74.4	87.9	+18.2%
Teamwork and project management score (out of 10)	7.2	8.6	+19.4%

The improvement was particularly evident among students who actively used the AI-based personalized learning system. Learning analytics revealed that adaptive feedback loops—providing real-time error detection and targeted review exercises—led to a significant reduction in repetitive coding mistakes and a more efficient learning trajectory. The average number of system-logged interactions per student increased by 34%, suggesting heightened engagement and deeper cognitive involvement in coursework.

Qualitative feedback further supports these quantitative trends. Over 85% of surveyed students reported that AI-driven diagnostic tools enhanced their conceptual understanding and confidence in tackling complex programming problems. Several students highlighted the role of intelligent tutors in promoting reflective learning; for instance, one participant remarked, “The AI assistant helped me identify gaps in logic during coding and recommended resources that fit my learning style.”

Additionally, participation in national and institutional competitions, such as the Blue Bridge Cup, the National College Student Innovation and Entrepreneurship Training Program (Dachuang), the National College Student Programming Contest (Big Challenge), the National College Student Programming Contest (Small Challenge), and various provincial-level competitions, has significantly increased. The number of student-led teams has risen by 40% over 2 years. Furthermore, the output of student-driven research papers and patent applications has also grown correspondingly, demonstrating a measurable enhancement in innovation literacy and practical competence.

### 4.2 Strengthening of teaching effectiveness

Teaching effectiveness scores were assessed using structured course evaluation questionnaires, which were distributed to students at the end of each course. The questionnaires were administered to both the pre-reform cohort and the post-reform cohort, corresponding to the same courses. A total of 205 students completed the survey in the pre-reform cohort, and 244 students completed the survey in the post-reform cohort. The evaluation framework encompassed three key dimensions: instructional clarity, feedback timeliness, and student interaction. All data were fully anonymized, collected in accordance with institutional ethical guidelines, and student participation in the surveys and course evaluations was entirely voluntary. No personally identifiable information was included in the dataset.

(1) Instructional clarity was measured using a five-point Likert scale, in which respondents rated the clarity, organization, and comprehensibility of course content, lecture delivery, and stated learning objectives.
(2) Feedback timeliness was assessed by asking students to rate the promptness and responsiveness of instructors in providing feedback on assignments, projects, and in-class activities.
(3) Student interaction was evaluated based on students’ perceptions of the frequency and quality of in-class discussions, group work engagement, and opportunities for collaborative learning.

For each dimension, scores were averaged across respondents and normalized to a 100-point scale. The overall teaching effectiveness score was calculated as the mean of the three dimension scores. Analysis over two consecutive semesters indicated an increase of 21% in overall teaching effectiveness, with improvements observed across all three dimensions (Table 3). Faculty members reported that the intelligent dashboard systems enabled them to identify struggling students early, personalize learning materials, and adapt instructional pacing more effectively.

**Table 3:** Comparison of teaching effectiveness scores before and after the educational reform.

Dimension	Pre-reform cohort (*n* = 205)	Post-reform cohort (*n* = 244)	Absolute change (points)	Relative change (%)
Instructional clarity	70	83	13	18.57%
Feedback timeliness	65	80	15	23.08%
Student interaction	65	79	14	21.54%
Overall teaching effectiveness	66.67	80.67	14	21.00%

AI integration has also transformed teachers’ pedagogical practices. Automated grading, attendance tracking, and learning diagnostics have substantially reduced administrative workload, allowing educators to focus more on mentoring and higher-order teaching activities. Smart classroom assistants equipped with speech recognition and sentiment analysis tools provide teachers with insights into classroom dynamics, helping them adjust communication strategies and maintain student engagement. As one instructor noted, “The AI dashboard lets me see which concepts students are struggling with in real time—something I could never do before.”

In addition, structured professional development programs have played a crucial role in building teachers’ AI literacy and pedagogical innovation capabilities. Over 65 faculty members have completed certification courses on AI pedagogy, learning analytics, and interdisciplinary curriculum design. These initiatives have cultivated a community of practice in which teachers share case studies, co-develop AI-enhanced lesson plans, and conduct action research on smart teaching models. Results from follow-up evaluations show that more than 75% of participants have incorporated AI tools into at least one course, leading to measurable gains in instructional efficiency and student satisfaction.

Moreover, the reform has fostered a culture of reflective and evidence-based teaching. Faculty members routinely analyze classroom data to refine teaching strategies, while institutional teaching research centers use aggregated analytics to support curriculum optimization. The establishment of “AI Teaching Innovation Teams” encourages cross-departmental collaboration and ensures the sustainability of pedagogical reform. Collectively, these developments signify a paradigm shift from experience-based to data-driven instruction, empowering teachers to deliver more adaptive, efficient, and student-centered learning experiences.

### 4.3 Enhanced integration of industry resources

The establishment of collaborative laboratories and training platforms with Huawei and Neusoft has substantially deepened the integration of industry resources into the teaching and learning process. Through these partnerships, students gain direct access to enterprise-grade technologies, professional mentorship, and authentic industrial projects that simulate real workplace scenarios. The joint laboratories serve not only as venues for technical experimentation but also as innovation incubators where students participate in R&D activities aligned with current industrial trends in AI, cloud computing, and cybersecurity.

To ensure close alignment between academic training and industry standards, both Huawei and Neusoft have been actively involved in course co-design and practical training supervision. Enterprise experts regularly deliver guest lectures, evaluate student projects, and provide professional guidance throughout the learning process. A dual-supervision model has been implemented in which each student undertaking a capstone project or internship is jointly mentored by a university instructor and an enterprise engineer. This model bridges theory and practice by allowing students to apply academic knowledge directly to enterprise-level problem-solving tasks, such as network security simulation, data analytics optimization, and intelligent system deployment.

Enterprise-level technical standards, platforms, and tools—such as Huawei’s cloud development environments and Neusoft’s software engineering toolkits—have been integrated into professional courses and project-based learning modules. These integrations expose students to cutting-edge development ecosystems and enhance their familiarity with industrial workflows and collaborative tools. In addition, joint innovation challenges and enterprise-sponsored project showcases have been organized annually, providing students opportunities to demonstrate AI-driven solutions to real industrial problems and to receive professional feedback from corporate mentors.

As a result, the alignment between curriculum content and industry demands has been significantly strengthened. Employer feedback consistently highlights graduates’ enhanced readiness for complex, real-world problem-solving, teamwork, and project management. Data from recent cohorts in the pilot programs indicate that both the employment rate and the quality of job placements have shown a steady upward trend, with a notable increase in positions secured at top-tier technology enterprises and R&D centers. Collectively, the collaboration with Huawei and Neusoft has established a sustainable mechanism for university–industry synergy, ensuring that talent cultivation remains responsive to technological evolution and labor market transformation.

## 5 Conclusion and future work

This study presents a comprehensive reform initiative aimed at enhancing the talent cultivation model for computer-related majors through the deep integration of artificial intelligence technologies within the framework of new quality productive forces. By redesigning curriculum structures, embedding intelligent tools across teaching processes, fostering interdisciplinary collaboration, and strengthening practice-based learning environments, the reform has made notable progress in aligning academic training with the evolving needs of the digital economy. Preliminary outcomes suggest that the AI-empowered approach may contribute to improvements in students’ technical proficiency, innovation capacity, and preparedness for complex, real-world challenges. Similarly, faculty members have reported perceived enhancements in instructional design and effectiveness, and institutional partnerships with industry stakeholders have appeared to support application-oriented education.

Despite these positive indications, several limitations should be noted. First, the study was conducted at a single institution, and industry collaboration primarily involved specific partners (Huawei and Neusoft), which may limit the generalizability of the findings. Second, the sample size of 244 students and data collection over two consecutive semesters may not fully capture long-term educational effects or students’ developmental trajectories. Third, while the proposed framework integrates curriculum restructuring, smart teaching, interdisciplinary collaboration, practice-oriented platforms, and multi-dimensional evaluation, its applicability to non-computer disciplines or across different institutions remains to be verified.

Future research will focus on expanding the reform model to additional majors and institutions, enhancing the adaptability of AI-enabled platforms to diverse learning contexts, and fostering a culture of continuous improvement through data-driven educational research. Furthermore, efforts will be made to deepen the integration of AI ethics, professional responsibility, and human-centered design into the computing curriculum, ensuring that students not only acquire technical excellence but also develop a strong sense of social responsibility and ethical awareness.

In sum, the AI-driven reform initiative presented in this study offers a replicable and scalable model for talent cultivation in the era of new quality productive forces. It contributes to the broader discourse on the digital transformation of higher education and provides actionable insights for institutions seeking to align their educational practices with future-oriented industry and societal demands.

## Data Availability

The data that support the findings of this study are available from the corresponding author upon reasonable request.

## Footnotes

1
https://www.huawei.com/en/trust-center/
